# Tianxiangdan Improves Coronary Microvascular Dysfunction in Rats by Inhibiting Microvascular Inflammation via Nrf2 Activation

**DOI:** 10.1155/2021/4114784

**Published:** 2021-12-02

**Authors:** Guligena Sawuer, Xue-Kuan Ma, Ya-Jie Zhang, Xuan-Ming Zhang, Zulihumaer Ainiwaer, Dong-Qing An

**Affiliations:** ^1^College of Traditional Chinese Medicine, Xinjiang Medical University, Urumqi 830011, China; ^2^Medical Department, The Fourth Affiliated Hospital of Xinjiang Medical University, Urumqi 830099, China; ^3^Department of Traditional Chinese Medicine, The First Affiliated Hospital of the Medical College, Shihezi University, Shihezi 832000, China; ^4^Xinjiang Key Laboratory of Famous Prescription and Science of Formulas, Xinjiang Medical University, Urumqi 830011, China

## Abstract

**Background:**

Tianxiangdan (TXD) is used in traditional Chinese medicine because of its therapeutic and preventive effects in the treatment of coronary heart disease. However, the underlying mechanism of TXD in coronary microvascular disease (CMD) remains unclear.

**Methods:**

A rat model of CMD was developed to study the mechanism of TXD activity. Sodium laurate was injected into the left ventricle of Sprague–Dawley rats to induce CMD. The rats were divided into six groups: a sham-operated (sham) group, an untreated CMD group, a low-dose TXD group (0.81 g·kg^−1^·d^−1^), a mid-dose TXD (TXD-M) group (1.62 g·kg^−1^·d^−1^), a high-dose TXD (TXD-H) group (3.24 g·kg^−1^·d^−1^), and a nicorandil (NCR) group (1.35 mg·kg^−1^·d^−1^). The effect of TXD on rats with CMD was observed after four weeks, and the mechanism of TXD in lipopolysaccharide (LPS)-induced cardiac microvascular endothelial cells (CMECs) was explored through treatment with 50 *μ*g/mL TXD.

**Results:**

Compared with the rats in the untreated CMD group, rats in the TXD-M and TXD-H groups showed higher left ventricular ejection fraction values, improved pathological structures, decreased expressions of interleukin (IL)-1*β*, tumor necrosis factor-alpha (TNF-*α*), phosphorylated nuclear factor-*κ*B inhibitor *α* (I*κ*B*α*) and phosphorylated p65, and increased expressions of nuclear factor erythroid 2-related factor 2 (Nrf2) and heme oxygenase-1 (*P* < 0.05). These effects were more pronounced in the TXD-H group than in the TXD-M group. *In vitro* experiments showed that TXD treatment increased the viability of LPS-induced CMECs and decreased the expression of IL-1*β*, TNF-*α*, phosphorylated I*κ*B*α*, and phosphorylated p65 (*P* < 0.05). However, the effects of TXD on CMECs were markedly reversed upon treatment with ML385 (Nrf2 inhibitor).

**Conclusion:**

The results showed that TXD exerts a protective effect on rats with CMD and related inflammatory injuries, and its anti-inflammatory mechanism is related to the activation of Nrf2 signalling.

## 1. Introduction

Coronary microvascular dysfunction (CMD), which is characterised by an abnormal structure and/or function of the coronary microvasculature (vessels with a diameter <500 *μ*m) [1], is a type of coronary heart disease (CHD). It is very common in patients with ischemia and nonobstructive coronary artery disease [2], with an incidence rate of approximately 60% [3]. However, current CMD treatments remain ineffective due to the disease's complex pathogenesis.

Inflammation is an important pathological process in CMD [4]; it can injure coronary microvessels to promote thrombosis and perivascular fibrosis. Endothelial cells are the main regulators of vascular inflammation [5]. According to previous studies, an injection of sodium laurate into coronary microvessels can promote microvascular endothelial injury and microthrombus formation [6, 7]. Moreover, lipopolysaccharide (LPS) can activate the nuclear factor-*κ*B (NF-*κ*B) pathway to upregulate inflammatory cytokines, such as tumor necrosis factor-alpha (TNF-*α*), interleukin (IL)-1 and IL-6 [8]. In contrast, the proinflammatory effect of LPS can be inhibited by the activation of nuclear factor erythroid 2-related factor 2 (Nrf2) [9]. Heme oxygenase-1 (HO-1), a downstream protein of the Nrf2 pathway, can decrease inflammation by inhibiting NF-*κ*B transcriptional activity [10, 11].

Tianxiangdan (TXD) granules, a Chinese herbal compound composed of *Rhodiola rosea*, *Ziziphora clinopodioides*, *Lignum dalbergiae odoriferae*, and *Salvia miltiorrhiza*, have been used to prevent and treat CHD for over 30 years [12]. TXD has been found to decrease the serum expression of IL-1*β* and TNF-*α* in patients with CHD [13] and decrease NF-*κ*B protein expression in mice with atherosclerosis [14]. A recent study conducted by the authors of the present study found that TXD can increase the Nrf2 and downstream SOD expressions as well as reduce the expression of MDA in rats with CMD [15, 16]. *R. rosea* reduces neuroinflammation by regulating the Nrf2/NF-*κ*B signalling pathway [17], and *S. miltiorrhiza* inhibits inflammatory responses and thrombosis in patients with thromboangiitis obliterans [18]. The results of the recent study suggest that TXD alleviates inflammation and ameliorates the clinical symptoms of CHD. However, the effects of TXD on CMD still remain unclear. The present study, therefore, aimed to investigate the underlying mechanism of the effects of TXD on CMD and determine its inhibitory effect on microvascular inflammation.

## 2. Materials and Methods

### 2.1. Medications

TXD granules were purchased from Xinjiang Huashidan Pharmaceutical Co., Ltd., (Xinjiang, China), and registered as a national patent under no. 200910210063.9 in 2009. TXD (9 g/bag; 3.125 g pure medicine/bag) is composed of *R. rosea*, *S. miltiorrhiza*, *Z. clinopodioides*, and *Lignum dalbergiae odoriferae* at a ratio of 3 : 3 : 2 : 1. Nicorandil tablets were obtained from Tisci Ai (Shanghai, China), pentobarbital sodium, sodium laurate, and LPS were obtained from Sigma-Aldrich (St. Louis, MO, USA), and ML385 was purchased from MedChemExpress LLC (Shanghai, China).

### 2.2. Animals

Sprague-Dawley rats (180 ± 20 g) were provided by the Animal Experiment Center of Xinjiang Medical University (animal license number: SYXK[Xin] 2016–007). All animal experiments were approved by the Animal Ethics Committee of Xinjiang Medical University (IACUC-20170222027).

### 2.3. Animal Experiments

A total of 58 rats were anaesthetised with 40 mg/kg pentobarbital sodium (10 mg/ml). The skin and subcutaneous tissue were cut longitudinally from the third and fourth left ribs to open the chest, and 1 ml/kg sodium laurate (2 mg/ml) was rapidly injected into the left ventricle. Simultaneously, the ascending aorta and aortic arch joint were clipped for 10 seconds with haemostatic forceps to ensure that sodium laurate flowed into the microvessels. The sham group underwent the same procedure, with the exception of the left ventricle receiving the same volume of normal saline instead. To determine the establishment of the CMD model, five model rats and five sham-operated rats were randomly selected and euthanised after 24 hours. The myocardial tissues of these rats were then excised to make tissue sections, and the hematoxylin and eosin (HE) stained myocardial tissue sections were observed under a light microscope. The rats with CMD who had microvascular endothelial injury and microthrombus formation were considered successful models [7].

The rats were then randomly divided into six groups: a sham group (*n* = 8; blank control), an untreated CMD group (*n* = 8), a low-dose TXD (TXD-L) group (*n* = 8), a mid-dose TXD (TXD-M) group (*n* = 8), a high-dose TXD (TXD-H) group (*n* = 8), and a nicorandil (NCR) group (*n* = 8; positive control). The rats underwent standard feeding under specific pathogen-free conditions with unrestricted activity. The TXD was administered to rats in the different groups based on the following formula:

(1) $$\begin{array}{c} \text{Daily TXD}-\text{M dose}=6.3\times \text{normal clinical dose for adults}\left(18\text{ g}\cdot {\text{d}}^{-1},\text{average adult weight was recorded as }70\text{ kg}\right)\\ \text{Daily TXD}-\text{L dose}=\frac{\text{dose of TXD}-\text{M}}{2};\\ \text{Daily TXD}-\text{H dose}=\text{dose of TXD}-\text{M}\times 2. \end{array}$$

Hence, the daily doses were as follows: TXD-L = 0.81 g·kg^−1^·d^−1^; TXD-M = 1.62 g·kg^−1^·d^−1^; and TXD-H = 3.24 g·kg^−1^·d^−1^. The NCR group was given nicorandil (1.35 mg·kg^−1^·d^−1^), while the sham group and the CMD groups were given normal saline (2 mL·d^−1^). The drugs were administered intragastrically once a day for four weeks. The rats were provided with ordinary diets and maintained at room temperature (20–24°C) and 40–70% relative humidity.

After four weeks of treatment, all rats were anaesthetised with 40 mg/kg pentobarbital sodium (10 mg/ml), and their cardiac functions were evaluated using echocardiography. Blood was then drawn from the abdominal aorta, and serum was collected. Rat myocardial tissues were taken for observation of pathologic structure and detection of protein expression in the myocardium.

### 2.4. Echocardiographic Assessment

Echocardiography was conducted four weeks after treatment using an ultrasonic diagnostic instrument (Philips, Amsterdam, Netherlands). An M-type echocardiograph was performed to continuously measure the left ventricular end-systolic and end-diastolic dimensions. Then, three cardiac cycles were repeated, and the mean values were calculated. The left ventricular ejection fraction (LVEF) was calculated according to the ejection fraction formula. These assessments were performed by a professional blinded to the groupings.

### 2.5. HE Staining

Rat myocardial tissues were excised and immediately immobilised with a tissue fixator for 48 hours. Then, they were dehydrated with different concentrations of ethanol, embedded in paraffin, cut into slices, and stained with HE. Inflammation severity was graded from 0 to 5 based on the extent of inflammatory cell infiltration: 0 = no infiltration; 1 ≤ 10%; 2 = 10–25%; 3 = 26–50%; 4 = 51–75%; and 5 ≥ 75% [19, 20].

### 2.6. Microvessel Density Calculation

Myocardial tissue sections were stained with anti-CD34 primary antibody (1 : 200, Abcam, Cambridge, UK). Vascular endothelial cells were immunohistochemically labeled with CD34 to calculate microvessel density (MVD) [21], which was quantified by counting the number of brown endothelial cells or endothelial cell clusters. Five microscopic fields were randomly selected for each tissue section under 400× magnification, and their average values were taken as the MVD of each sample.

### 2.7. Ultrastructure Analysis Using Transmission Electron Microscopy

Myocardial tissues were cut into approximately 1 × 1 mm^3^ blocks on ice, fixed, embedded, and sliced into ultrathin sections. Ultrastructural changes in myocardial microvessels were examined under a transmission electron microscope (Hitachi, Tokyo, Japan) using an 80 kV accelerating voltage and an amplification rate of 10,000.

### 2.8. Cell Culture and Treatment

Human cardiac microvascular endothelial cells (CMECs) were obtained from Zhongqiao Xinzhou Biotechnology Co., Ltd. (Shanghai, China) and cultured at 37°C in an endothelial cell medium (ScienCell, Carlsbad, CA, USA) in a 5% CO_2_ incubator. The optimal concentrations of TXD and LPS were determined using a cell viability assay. The CMECs were then treated with 5 *μ*M of ML385 (Nrf2 inhibitor) to determine whether TXD inhibits inflammation by activating Nrf2 [22], after which they were seeded into 6-well plates at a density of 1 × 10^5^/well. The cells reached 80% confluency following overnight incubation. These cells were treated as follows: a control group (normal medium), an LPS group (10 *μ*g/mL LPS for 24 hours), an LPS + TXD group (50 *μ*g/mL TXD +10 *μ*g/mL LPS for 24 hours), and an ML385 + LPS + TXD group (5 *μ*M ML385 pretreatment for 12 hours, followed by 50 *μ*g/mL TXD +10 *μ*g/mL LPS for 24 hours).

### 2.9. Cell Viability Assay

The viability of the CMECs was determined using an MTT assay, after which they were seeded into a 96-well plate at a density of 1 × 10^4^/well. After reaching 80% confluency during overnight incubation, the cells were treated as aforementioned, and cell viability was measured at 0, 12, 24, 36, and 48 hours after treatment. MTT reagent (20 *μ*L) was added to each well, and the plate was cultured at 37°C for 4 hours. The cell supernatant was then removed from each well, and the cells were treated with 150 *μ*L dimethyl sulfoxide. The plate was shaken for 200 seconds, and cell viability was measured at 490 nm using a microplate reader.

### 2.10. Immunofluorescence Staining

CMECs were seeded into confocal dishes at a density of 2 × 10^4^/dish and incubated overnight at 37°C. The CMEC expressions of Nrf2 and NF-*κ*B p65 were determined by immunofluorescence staining. The cells were then briefly fixed and incubated overnight at 4°C with the following primary antibodies: anti-Nrf2 (1 : 200, Abcam) and anti-NF-*κ*B p65 (1 : 500, Abcam). The next day, the cells were treated with the corresponding secondary antibodies at room temperature (20–27°C) for 2 hours. Finally, the cells were counterstained with 4′,6-diamidino-2-phenylindole (Solarbio, Beijing, China) for 10 minutes and visualised using confocal microscopy.

### 2.11. Enzyme-Linked Immunosorbent Assay

An enzyme-linked immunosorbent assay was used to measure the serum IL-1*β* and TNF-*α* expressions in the CMECs and rats with CMD. The assay kit was purchased from Jianglai Biological Ltd. (Shanghai, China), and all experiments were performed in accordance with the manufacturer's instructions.

### 2.12. Western Blotting

The total protein extracted from the rat myocardial tissues and CMECs was electrophoresed and transferred to a polyvinylidene difluoride membrane. The membrane was blocked with 5% skim milk powder solution for 2 hours and incubated overnight at 4°C with the following primary antibodies: anti-Nrf2 (1 : 1000), anti-HO-1 (1 : 2000), anti-I*κ*B*α* (1 : 2500), anti-p-I*κ*B*α* (1 : 500), anti-NF-*κ*B p65 (1 : 2000), anti-p-p65 (1 : 2000), anti-TNF-*α* (1 : 2000), anti-IL-1*β* (1 : 2000), and anti-*β*-actin (1 : 5000). The next day, the membrane was incubated with the corresponding secondary antibodies at room temperature for 2 hours. Finally, protein bands were visualised and analysed using the Bio-Rad image lab software (Hercules, CA, USA). All antibodies except anti-p-I*κ*B*α* (Santa Cruz Biotechnology, Dallas, TX, USA) were purchased from Abcam.

### 2.13. Statistical Analysis

Data were analysed using GraphPad Prism (version 8.0; GraphPad, Inc., La Jolla, CA, USA). Based on the results of a normality test and a homogeneity test of variance, a one-way analysis of variance or rank-sum test was used to compare differences between groups. The Tukey test was used if the data conformed to the normal distribution and the variance was homogeneous, while the Brown–Forsythe test and Welch test were used if the data did not conform to the normal distribution or the variance was heterogeneous. *P* < 0.05 was considered statistically significant.

## 3. Results

### 3.1. TXD Improved Cardiac Function in Rats with CMD

**Figure 1:**
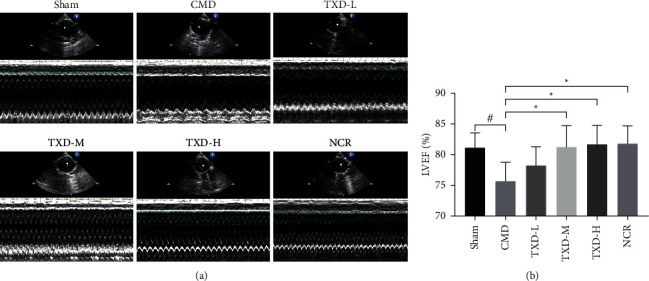
TXD ameliorated cardiac function in CMD rats. (a) Representative echocardiographic images of all rat groups. (b) LVEF of each group. ^#^*P* < 0.05 vs. sham group; ^*∗*^*P* < 0.05 and ^*∗∗*^*P* < 0.01 vs. CMD group. TXD, tianxiangdan granules; CMD, coronary microvascular dysfunction; LVEF, left ventricular ejection fraction; Sham, sham group; CMD, CMD model group; TXD-L, TXD low-dose (0.81 g·kg^−1^·d^−1^) group; TXD-M, TXD mid-dose (1.62 g·kg^−1^·d^−1^) group; TXD-H, TXD high-dose (3.24 g·kg^−1^·d^−1^) group; NCR, nicorandil (1.35 mg·kg^−1^·d^−1^) group.

To evaluate cardiac function, the LVEF values were measured using echocardiography. The LVEF value was significantly lower in the untreated CMD group (75.68 ± 3.09%) than in the sham group (81.14 ± 2.43%) (*P* < 0.05), suggesting a decreased cardiac function in rats with CMD. Furthermore, the LVEF values were significantly higher in the TXD-M group (81.26 ± 3.44%) and the TXD-H group (81.72 ± 3.04%) than in the CMD group (*P* < 0.05), suggesting that treatment with TXD improved cardiac function in rats with CMD (see Figure 1).

### 3.2. TXD Improved the Myocardial Microvascular Pathological Structures of Rats with CMD

**Figure 2:**
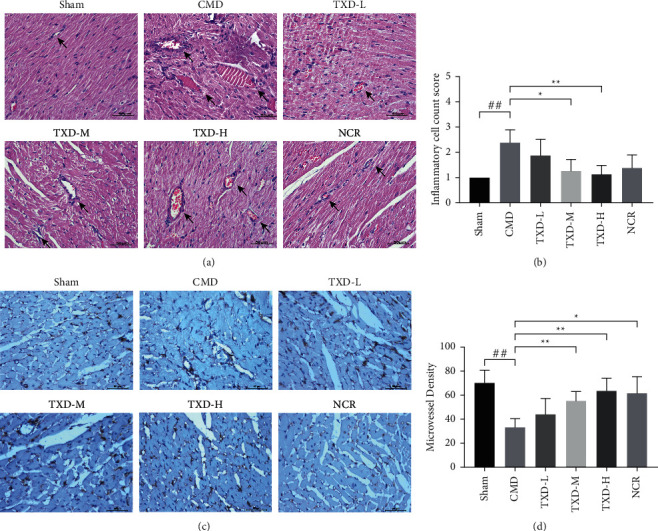
TXD improved the arrangement of myocardial microvascular pathologic structures and increased MVD in CMD rats. (a) Representative histological images of HE-stained myocardial sections (×400). Arrows indicate microvessels. (b) Inflammatory cell count score of each treatment group. (c) Immunohistochemical staining of the myocardium with anti-CD34 antibody (×400). Vascular endothelial cells are stained as pale brown; arrows indicate microvessels. (d) Determination of MVD. ^##^*P* < 0.01 vs. sham group; ^*∗*^*P* < 0.05 or ^*∗∗*^*P* < 0.01 vs. CMD group. HE, hematoxylin-eosin; MVD, microvascular density; TXD, tianxiangdan granules; TXD-L, low-dose TXD; TXD-M, mid-dose TXD; TXD-H, high-dose TXD; CMD, coronary microvascular dysfunction; NCR, nicorandil.

To evaluate the morphological and quantitative changes in the myocardial microvessels of rats with CMD, the myocardial and microvascular pathological structures were observed using HE staining, after which MVD was detected by calculating immunohistochemical CD34-labeled microvessels. Disordered myocardial fibers with microvascular thrombus formations were observed in the untreated CMD group. In comparison with the sham group, the mean inflammatory cell infiltration score (2.38) and MVD score (33.17) in the untreated CMD group were significantly different (*P* < 0.01). Myocardial pathological structures and MVD did not significantly improve in the TXD-L group, but the TXD-M and TXD-H groups showed improved myocardial fiber arrangement. Moreover, no microvascular thrombosis was observed in these groups. The mean inflammatory cell infiltration scores and MVD of the TXD-M group (1.25 and 55.17) and the TXD-H group (1.13 and 64.67) were also significantly different than those of the CMD group (*P* < 0.05) (see Figure 2).

**Figure 3:**
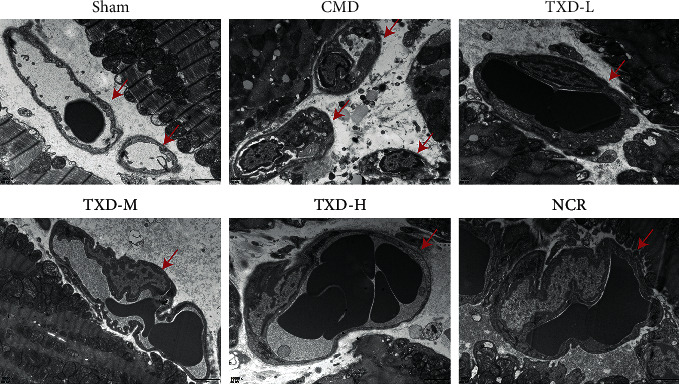
TXD improved myocardial microvascular ultrastructure in CMD rats. Electron microscopy images of capillary microstructures (×10,000). Red arrows indicate capillaries. TXD, tianxiangdan granules; TXD-L, low-dose TXD; TXD-M, mid-dose TXD; TXD-H, high-dose TXD; CMD, coronary microvascular dysfunction; NCR, nicorandil.

The ultrastructure of the myocardial microvasculature was also observed under an electron microscope. Thickened cytoplasm, widened perinuclear space, thrombus formation in capillaries, and swollen capillary endothelial cells were detected in the untreated CMD group. No swelling of capillary endothelial cells was observed in the TXD-M group or the TXD-H group, and only a slightly wrinkled basement membrane with no luminal thrombi was detected in these specimens (see Figure 3).

### 3.3. TXD Decreased IL-1*β*, TNF-*α*, p-I*κ*B*α*, and p-p65 Expression and Increased Nrf2 and HO-1 Expression in Rats with CMD

**Figure 4:**
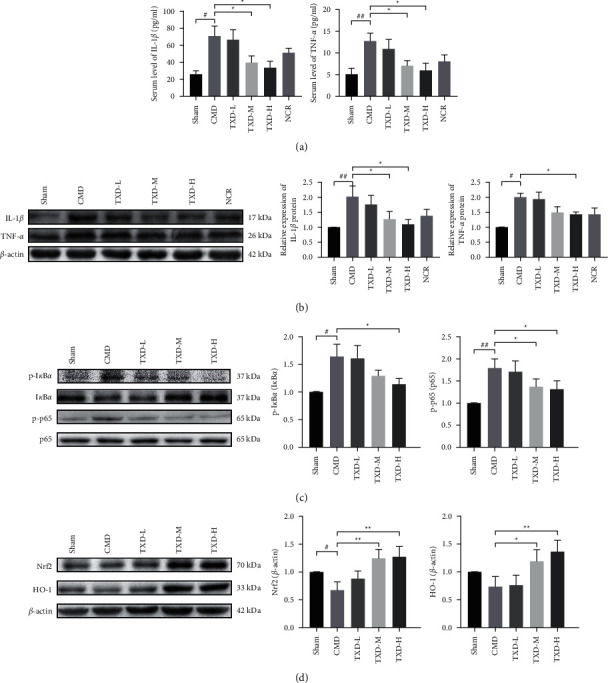
TXD decreased IL-1*β*, TNF-*α*, p-I*κ*B*α*, and p-p65 expression and increased Nrf2 and HO-1 expression in CMD rats. (a) Serum levels of IL-1*β* and TNF-*α*; (b) Representative protein bands of IL-1*β* and TNF-*α* and relative protein expression of IL-1*β* and TNF-*α* in CMD rats. (c) Representative protein bands of I*κ*B*α*, p-I*κ*B*α*, NF-*κ*B p65, and p-p65 and relative protein expression of p-I*κ*B*α* and p-p65 in CMD rats. (d) Representative protein bands of Nrf2 and HO-1 and relative expression in CMD rats. ^#^*P* < 0.05 or ^##^*P* < 0.01 vs. sham group; ^*∗*^*P* < 0.05 or ^*∗∗*^*P* < 0.01 vs. CMD group. IL-1*β*, interleukin 1*β*; TNF-*α*, tumor necrosis factor-*α*; I*κ*B*α*, NF-kappa-B inhibitor alpha; p-I*κ*B*α*, phosphorylated NF-kappa-B inhibitor alpha; NF-*κ*B p65, nuclear factor-kappa B subunit p65; p-p65, phosphorylated p65; Nrf2, nuclear factor erythroid 2-related factor 2; HO-1, heme oxygenase-1; TXD, tianxiangdan granules; TXD-L, low-dose TXD; TXD-M, mid-dose TXD; TXD-H, high-dose TXD; CMD, coronary microvascular dysfunction; NCR, nicorandil.

To determine the extent of microvascular inflammation, the serum expressions of IL-1*β* and TNF-*α* and the myocardial tissue protein expressions of IL-1*β*, TNF-*α*, p-I*κ*B*α*, and p-p65 were measured in all groups. It was found that the serum expressions of IL-1*β* and TNF-*α*, as well as the protein expressions of IL-1*β*, TNF-*α*, p-I*κ*B*α*, and p-p65 were significantly higher in the untreated CMD group than in the sham group. However, the serum expressions of IL-1*β* and TNF-*α* were significantly lower in the TXD-M group and the TXD-H group than in those of the CMD group (*P* < 0.05). Furthermore, the protein expressions of p-p65 and IL-1*β* in the TXD-M group and the expressions of p-I*κ*B*α*, p-p65, IL-1*β*, and TNF-*α* in the TXD-H group were significantly lower than those in the CMD group (*P* < 0.05) (see Figures 4(a), 4(b), and 4(c)). To further understand the anti-inflammatory mechanism of TXD, the expressions of Nrf2 and HO-1 proteins in rats with CMD were determined. It was discovered that the protein expression of Nrf2 was significantly lower in the untreated CMD group than in the sham group and that the protein expressions of Nrf2 and HO-1 were higher in the TXD-M group and the TXD-H group than in the CMD group (*P* < 0.05) (see Figure 4(d)).

### 3.4. TXD Inhibited LPS-Induced CMEC Inflammation via Nrf2 Activation

**Figure 5:**
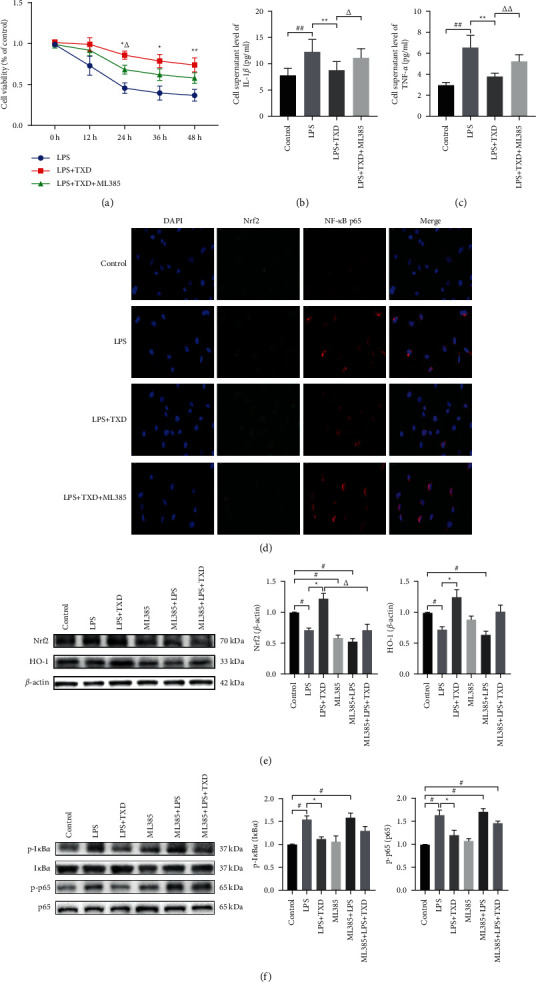
TXD inhibited LPS-induced CMEC inflammation via activation of Nrf2. (a) Viability of CMECs after treatment with 50 *μ*g/mL TXD and 10 *μ*g/mL LPS, as well as pretreatment with 5 *μ*M ML385 (Nrf2 inhibitor); (b and c) IL-1*β* and TNF-*α* expression in CMECs; (d) Immunofluorescence analysis of Nrf2 and NF-*κ*B p65 expression in CMECs, scale bar = 20 *μ*m; (e) Representative protein bands of Nrf2 and HO-1 and relative expression levels in CMECs; (f) Representative protein bands of I*κ*B*α*, p-I*κ*B*α*, NF-*κ*B p65, and p-p65, and relative protein expression of p-I*κ*B*α* and p-p65, in CMECs. ^#^*P* < 0.05 or ^##^*P* < 0.01 vs. control group; ^*∗*^*P* < 0.05 or ^*∗∗*^*P* < 0.01 vs. LPS group; ^Δ^*P* < 0.05 or ^ΔΔ^*P* < 0.01 vs. LPS + TXD group. TXD, tianxiangdan granules; CMECs, cardiac microvascular endothelial cells; LPS, lipopolysaccharide.

The optimal concentration of TXD and LPS cotreated CMECs was determined by measuring cell viability using an MTT assay. The viability was significantly lower after 12 hours in the CMECs treated with 10 *μ*g/mL LPS than in the control group (*P* < 0.05). Furthermore, <50% of LPS-stimulated CMECs remained viable after 24 hours. The viability of the CMECs cotreated with 10 *μ*g/mL LPS and 50 *μ*g/mL TXD significantly increased, reaching a maximum at 24 hours (*P* < 0.05). However, when the CMECs were pretreated with ML385 (5 *μ*M) for 12 hours before the aforementioned treatment, the protective effect of TXD was weakened; this was most noticeable at 24 hours (*P* < 0.05) (see Figure 5(a)).

Based on these *in vivo* experimental results, it can be suggested that TXD exerts an anti-inflammatory effect on CMECs by regulating the Nrf2 pathway. Notably, the expressions of IL-1*β* and TNF-*α* were lower in cells cotreated with TXD and LPS than in cells treated only with LPS (*P* < 0.05). However, the expressions of IL-1*β* and TNF-*α* were not decreased by TXD when the CMECs were pretreated with ML385 (*P* < 0.05) (see Figures 5(b) and 5(c)).

The immunofluorescence results showed increased Nrf2 expression and decreased NF-*κ*B p65 expression in the cells cotreated with TXD and LPS. However, in the ML385-pretreated cells after cotreatment with TXD and LPS, Nrf2 expression decreased and p65 expression increased (see Figure 5(d)). The western blotting results further verified that TXD treatment increased Nrf2 and HO-1 protein expressions in LPS-induced CMECs (*P* < 0.05). However, the TXD-induced protein expressions of Nrf2 and HO-1 decreased upon the pretreatment of LPS-induced CMECs with ML385. At the same time, treatment of LPS-induced CMECs with TXD decreased the expression of p-I*κ*B*α* and p-p65 (*P* < 0.05). ML385 pretreatment increased the expression of p-I*κ*B*α* and p-p65 in TXD-treated LPS-induced CMECs. These results indicate that Nrf2 is important for TXD-induced inactivation of the NF-*κ*B pathway.

## 4. Discussion

CMD is a common type of CHD that can cause angina pectoris and myocardial ischemia due to structural or functional abnormalities in coronary microvessels [23]. In recent years, CMD has been shown to be closely linked with CHD and other cardiovascular diseases. Therefore, it is important to understand the pathogenesis and treatment of CMD [24].

Studies have revealed that inflammation can lead to microvascular injury via the upregulation of proinflammatory factors, such as IL-1*β*, IL-6, and TNF-*α* [4, 25, 26]. Furthermore, inflammation can reduce myocardial contractility by injecting the microvascular endothelium [21], and microvascular injury and capillary rarefaction can decrease myocardial perfusion and impair cardiac function [27]. In the present study, sodium laurate was injected into the coronary arteries of rats with CMD to impair cardiac function. The myocardial and microvascular structures of the rats were damaged, which is consistent with the modelling results of previous studies [6]. In addition, the expressions of IL-1*β* and TNF-*α* were significantly increased in rats with CMD, suggesting the occurrence of inflammatory damage. Under normal conditions, NF-*κ*B is inhibited by I*κ*B and remains inactive. However, I*κ*B and NF-*κ*B are phosphorylated by subsequent stimulation, further activating inflammatory mediators [28]. Nrf2 activation is known to exert anti-inflammatory activity [29], and increased Nrf2 expression causes HO-1 activation and inhibits NF-*κ*B nuclear translocation [10, 30].

Traditional Chinese medicine has been effectively used as an alternative and complementary method for improving the clinical symptoms of CMD [31]. It works on the principle of the symptoms caused by CMD being a manifestation of *qi*–blood disharmony [32]. The replenishment of *qi* and the activation of blood circulation can harmonize the *qi* and the blood, thereby improving CMD symptoms. Studies have shown that pills of QiShen YiQi, a Chinese herbal compound for replenishing *qi* and activating the blood, thereby ameliorating myocardial microcirculatory disturbance by improving myocardial energy metabolism and inhibiting oxidative stress injury in microcirculatory vasculature [33]. It has been suggested that the approach of replenishing *qi* and activating the blood has a therapeutic effect on CMD.

TXD, which is also a Chinese herbal compound for replenishing *qi* and activating the blood, is composed of *R. rosea*, *S. miltiorrhiza*, *Z. clinopodioides*, and *Lignum dalbergiae odoriferae. R. rosea*, which primarily contains salidroside, flavones, and volatiles, has been confirmed to have protective effects in the treatment of inflammatory injury [34]. Salidroside specifically has been found to activate the Nrf2-ARE signal pathway and suppress oxidative stress in rats [35]. *S. miltiorrhiza* is regarded as a “golden herb” in cardiovascular therapeutics; its critical pharmacological component is tanshinone [36], and it has been found that tanshinone IIA alleviates cardiac microvascular ischemia-reperfusion injury via SIRT1/PGC1*α* pathway activation [37]. In China, *Z. clinopodioides* is mainly produced in the semiarid region of Xinjiang. It is widely used by local people to treat cardiovascular and cerebrovascular diseases, and its flavonoid constituents protect myocardial cells from ischemia-reperfusion injury by removing oxygen-free radicals [38]. *Lignum dalbergiae odoriferae* is primarily composed of volatile oils and flavonoids. It has also been documented to exhibit antithrombotic, anti-inflammatory, and antioxidant biological effects [39]. The abovementioned studies fully demonstrate that TXD has cardioprotective and anti-inflammatory effects; however, its protective effect on CMD remains unclear.

The present study focused on the group effect of a compound mixture instead of a single agent. It found that TXD improved cardiac function, increased microvessel density, improved myocardial microvascular pathological structure organisation, and inhibited inflammation in rats with CMD. These effects were more pronounced after high-dosage TXD treatment (3.24 g·kg^−1^·d^−1^), and the results indicate that TXD exerts a protective effect in rats with CMD. The present study also found that TXD downregulated the expressions of p-I*κ*B*α*, p-p65, IL-1*β*, and TNF-*α* and upregulated the expressions of Nrf2 and HO-1 in LPS-induced CMECs and rats with CMD. Therefore, TXD may exert its protective effects on rats with CMD by promoting anti-inflammatory responses. To further understand the mechanism by which TXD improves microvascular inflammation, LPS-induced CMECs were treated with TXD. Notably, it was found that TXD had a protective effect on LPS-induced CMECs and suppressed the expressions of p-I*κ*B*α*, p-p65, IL-1*β*, and TNF-*α* by activating Nrf2. These results indicate that Nrf2 is important for the TXD-induced inhibition of microvascular inflammation.

## 5. Conclusion

**Figure 6:**
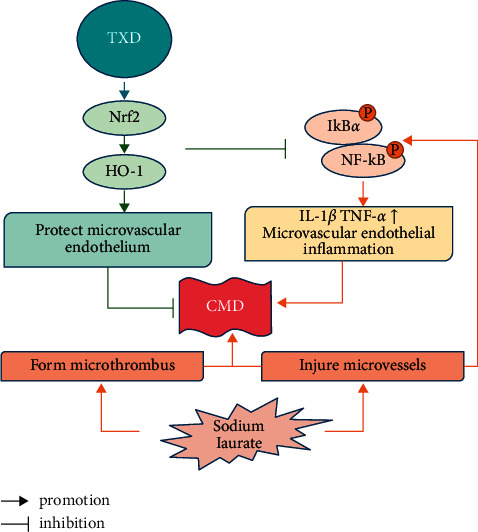
TXD inhibits microvascular inflammation via activation of Nrf2 in CMD rats. TXD, tianxiangdan granules; CMD, coronary microvascular dysfunction.

The present study shows that TXD exerts protective effects on rats with CMD and related inflammatory injury and that a high-dosage TXD treatment had the most pronounced effects. The inhibitory effect of TXD on microvascular endothelial inflammation is related to Nrf2 activation (see Figure 6). Therefore, the present study provides an experimental basis for the effectiveness of TXD in CMD treatment. TXD is a promising therapeutic candidate for treating coronary microvascular diseases.

## Data Availability

The datasets used to support the findings of this study are included within the article

## Supplementary Materials

Supplementary Materials Graphical abstract.
