# Supplementary figures and images for: Tianxiangdan Improves Coronary Microvascular Dysfunction in Rats by Inhibiting Microvascular Inflammation via Nrf2 Activation
Source: Evid Based Complement Alternat Med. 2021 Dec 2;2021:4114784. doi: 10.1155/2021/4114784 (PMC8660204; doi:10.1155/2021/4114784)

**
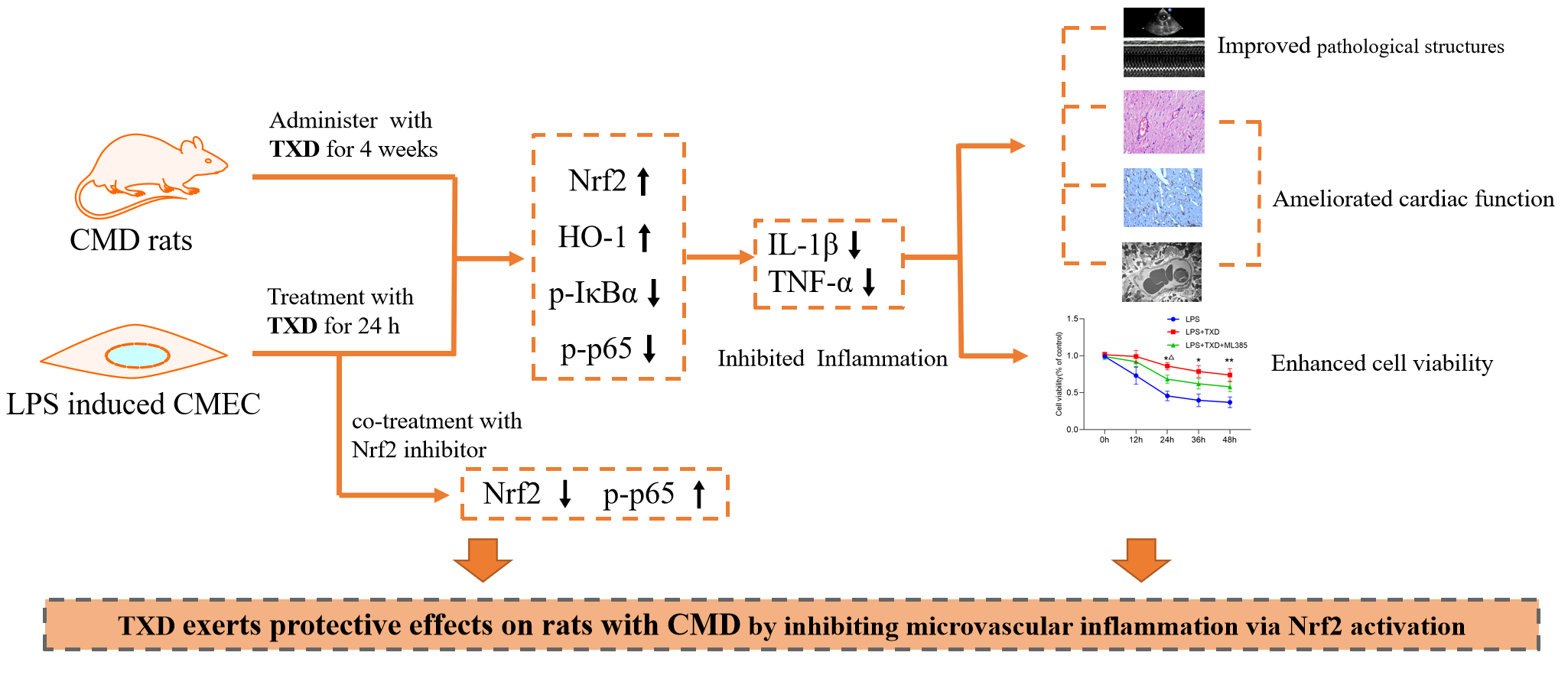
**

Supplement: Supplementary Materials — Graphical abstract. [file 4114784.f1.docx]
